# Placental structure, function, and mitochondrial phenotype relate to fetal size in each fetal sex in mice^†^

**DOI:** 10.1093/biolre/ioac056

**Published:** 2022-03-16

**Authors:** Esteban Salazar-Petres, Daniela Pereira-Carvalho, Jorge Lopez-Tello, Amanda Nancy Sferruzzi-Perri

**Affiliations:** Department of Physiology, Development and Neuroscience, Centre for Trophoblast Research, University of Cambridge, Cambridge, UK; Department of Physiology, Development and Neuroscience, Centre for Trophoblast Research, University of Cambridge, Cambridge, UK; Department of Physiology, Development and Neuroscience, Centre for Trophoblast Research, University of Cambridge, Cambridge, UK; Department of Physiology, Development and Neuroscience, Centre for Trophoblast Research, University of Cambridge, Cambridge, UK

**Keywords:** placenta, fetus, sex, mitochondria, transport, hormones

## Abstract

Fetal growth depends on placental function, which requires energy from mitochondria. Here we investigated whether mitochondrial function in the placenta relates to the growth of the lightest and heaviest fetuses of each sex within the litter of mice. Placentas from the lightest and heaviest fetuses were taken to evaluate placenta morphology (stereology), mitochondrial energetics (high-resolution respirometry), mitochondrial regulators, nutrient transporters, hormone handling, and signaling pathways (qPCR and Western blotting). We found that mitochondrial complex I and II oxygen consumption rate was greater for placentas supporting the lightest female fetuses, although placental complex I abundance of the lightest females and complexes III and V of the lightest males were decreased compared to their heaviest counterparts. Expression of mitochondrial biogenesis (*Nrf1*) and fission (*Drp1* and *Fis1*) genes was lower in the placenta from the lightest females, whilst biogenesis-related gene *Tfam* was greater in the placenta of the lightest male fetuses. In addition, placental morphology and steroidogenic gene (*Cyp17a1* and *Cyp11a1*) expression were aberrant for the lightest females, but glucose transporter (*Slc2a1*) expression was lower in only the lightest males versus their heaviest counterparts. Differences in intra-litter placental phenotype were related to changes in the expression of hormone-responsive (androgen receptor) and metabolic signaling (AMPK, AKT, and PPARγ) pathways. Thus, in normal mouse pregnancy, placental structure, function, and mitochondrial phenotype are differentially responsive to the growth of the female and male fetus. This study may inform the design of sex-specific therapies for placental insufficiency and fetal growth abnormalities with life-long benefits for the offspring.

## Teaser

Placenta structure, function, and mitochondrial functional capacity relate to the growth of the lightest and heaviest fetuses within the litter, and the nature of these changes differ in the two fetal sexes.

## Graphical Abstract

**Graphical Abstract:**
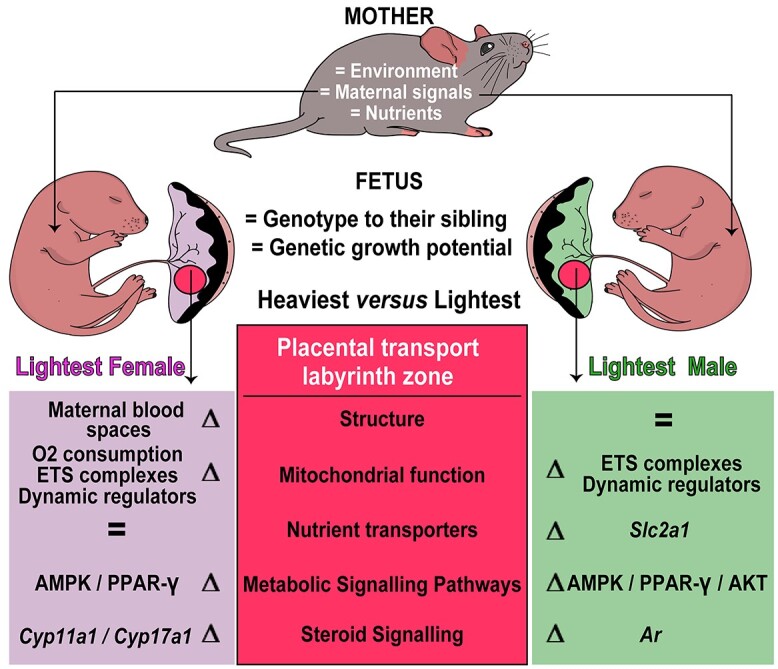

Here, we showed that in normal mouse pregnancy, placenta function varies between the lightest and the heaviest female and male fetuses within the litter. In particular, there are differences in mitochondria function, structure, nutrient transporters, metabolic pathways, and steroid signaling in the placental transport zone (labyrinth zone) that relate to fetal growth in the litter.

## Introduction

A successful pregnancy strongly depends on balancing resource allocation between the genetically determined fetal drive for growth and the mother who needs resources to support the pregnancy state. As a functional interface between mother and fetus, the placenta plays a key role in balancing fetal and maternal resource needs. Amongst its plethora of functions, the placenta executes the metabolism and secretion of hormones that have physiological effects on the mother and fetus and transfers nutrients and oxygen from the mother to the fetus [1]. Thus, it is perhaps unsurprising that fetal weight is related to placental development, the uteroplacental blood supply of nutrients and oxygen, and the capacity of the placenta to transport substrates to the fetus [2–8]. Moreover, failure of the placenta to grow and function properly is associated with the divergence of the fetus from their genetic growth potential and can lead to small for gestational age (SGA) and fetal growth restriction (FGR) [9–11]. Large for gestational age (LGA) can also occur during diabetic pregnancies with placentas showing hypervascularization, the elevation of angiogenic signals, altered metabolic genes, and oxidative stress [12]. SGA, FGR, and LGA not only increase the risk of perinatal morbidity and mortality but also have long-term consequences for offspring health [13]. Thus, it is important to understand the placental mechanisms regulating fetal growth outcomes.

To enable normal placental growth and function, the placenta depends on the energy supplied by mitochondria. Mitochondria are the primary source of ATP, which is produced by oxidative phosphorylation (OXPHOS) using substrates derived from β-oxidation and the tricarboxylic acid cycle. ATP is used by the placenta to fuel growth and placental endocrine and transport functions. Mitochondria are also the place within the cell where steroidogenesis occurs; they contain several key proteins and enzymes such as steroidogenic acute regulatory protein (STAR) and cholesterol side-chain cleavage enzyme (CYP11A1), which are required for glucocorticoid and sex-steroid synthesis [14]. Mitochondria are also involved in cell signaling, homeostasis, and survival via the production of reactive oxygen species and other molecules like nitric oxide. They are also dynamic organelles that can replicate (biogenesis), divide (fission), and combine (fusion) in response to metabolic, growth, and stress signals [15, 16]. During pregnancy, there are temporal changes in placental mitochondrial respiratory capacity and mitochondrial-related proteins in several species [15, 17–20]. Increasing evidence also suggests that placental mitochondrial function (mitochondrial OXPHOS, abundance, biogenesis, fission-fusion, and efficiency) alters in line with defects in fetal growth and placental development in response to experimental reductions in maternal nutrient and oxygen availability [21–24]. However, little is known about the relationship between placental mitochondrial capacity, placental morphological development, and natural deviations in fetal growth in normal, uncompromised pregnancies. Even less is known about whether this relationship may vary for female and male fetuses, which is highly relevant given that sex is emerging as an important contributor to changes in placental, fetal, and offspring health outcomes [25, 26].

In this study, we employed an integrative approach to evaluate placental morphology, mitochondrial OXPHOS capacity and mitochondrial regulator expression (ETS, electron transport system) complexes and biogenesis and fission-fusion regulators), in relation to the growth of the lightest and heaviest female and male fetuses within the litter of normal wildtype mice. Importantly, since the mouse is a polytocous species, normal variation of fetal weight is expected within the litter, even in a normal, healthy gestational environment. We also examined the activity of signaling pathways governing placental growth and metabolism, as well as the expression of nutrient transporter and steroid hormone handling genes to further understand how placental phenotype is modulated by fetal weight for each sex within the litter. Analyses were conducted on the labyrinth zone (Lz) of the mouse placenta as it is responsible for controlling the transport of nutrients, oxygen, and hormones from mother to fetus.

## Methods

### Animals

All experiments were performed under the U.K. Animals (Scientific Procedures) Act 1986 after ethical approval by the University of Cambridge. A total of 13 C57BL/6 J virgin female mice were housed in the University of Cambridge Animal Facility using a 12/12 dark/light system and received *ad libitum* water and chow food (Rodent No. 3, breeding chow; Special Diet Services, Witham) during the study. At 4 months of age, females were mated overnight and the day a copulatory plug was found was designated as the gestational day (GD) 1. On GD18, pregnant dams were killed by cervical dislocation, uteri were recovered, and fetuses and placentas were cleaned from fetal membranes. All fetuses and placentas from the litter were weighed. Litter size ranged from 6 to 10 pups, with a mean ± SEM of 8.00 ± 0.34 fetuses and overall, the percentage of female and male fetuses was 48.3% and 51.7%, respectively (Supplementary Table SS1). For seven litters, the placental Lz was micro-dissected, to separate it from the placental endocrine junctional zone and maternal decidua [27]. Lz samples were then cut in half, and one Lz half was placed into an ice-cold biopsy preservation medium (0.21 M Mannitol, 0.07 M Sucrose, 30% DMSO in H_2_O and pH 7.5) and frozen at −80 °C until respirometry analysis. The remaining half of the Lz was snap-frozen and stored at −80 °C for molecular analyses (RT-qPCR and Western Blot). Placental samples from the additional five litters were kept whole, placed into 4% paraformaldehyde, histologically processed, and used for structural analysis. The fetal brain and liver were dissected and weighed from each fetus. Fetal tails were kept for sex determination by detection of the *Sry* gene using the Taq Ready PCR system (Sigma), specific primers (*Sry*: FPrimer: 5′-GTGGGTTCCTGTCCCACTGC-3′, RPrimer: 5′-GGCCATGTCAAGCGCCCCAT-3′ and PCR autosomal gene control: FPrimer: 5′-TGGTTGGCATTTTATCCCTAGAAC-3′, RPrimer: 5′-GCAACATGGCAACTGGAAACA-3′) and agarose gel electrophoresis.

### Placental Lz mitochondrial respiratory capacity

High-resolution respirometry (Oxygraph 2 k respirometer; Oroboros Instruments, Innsbruck, Austria) was used to assess the capacity for respiratory substrate use and ETS function. Cryopreserved Lz samples (10–15 mg) were gently thawed in ice-cold sucrose solution (0.25 M sucrose, 0.01 M TRIS–HCl, pH 7.5). Samples were then permeabilized in respiratory medium BIOPS (10 mM CaEGTA buffer, 0.1 μM free Ca^2+^, 1 mM free Mg^2+^, 20 mM imidazole, 20 mM taurine, 50 mM K-MES, 0.5 mM dithiothreitol, 6.56 mM MgCl2, 5.77 mM ATP, and 15 mM phosphocreatine, pH 7.1) containing saponin (5 mg in 1 ml, Sigma-Aldrich, UK) for 20 min on ice. Samples were then washed by three 5-min washes in respiratory medium MiR05 (0.5 mM EGTA, 3 mM MgCl_2_x6H_2_O, 20 mM taurine, 10 mM KH_2_PO_4_, 20 mM Hepes, 1 mg/mL of BSA, 60mM K-lactobionate, 110 mM sucrose, pH 7.1) in ice to remove all endogenous substrates and contaminants. Oxygen concentration (μM) and flux per tissue mass (pmol O_2_/s/mg) were recorded in real-time using calibrated oxygen sensors and Datlab software (Oroboros Instruments, Austria). Respiratory rates were corrected for instrumental background by DatLab, considering oxygen consumption of the oxygen sensor and oxygen diffusion out of or into the oxygraph chamber measured under experimental conditions in miR05 medium without any tissue present.

A substrate-inhibitor titration protocol was performed under the presence of octanoylcarnitine and using approximately 10–15 mg of permeabilized Lz tissue placed into each oxygraph chamber. Briefly, complex I substrate malate (2 mM) was added first to determine LEAK respiration (uncoupled from ATP synthesis; complex I LEAK or CI_Leak_). Next, ADP (5 mM), pyruvate (20 mM), and glutamate (10 mM) were added to obtain complex I oxygen flux under OXPHOS state (CI_P_). Then, succinate (10 mM) was added to provoke complex I and II dependent OXPHOS (CI + ÍI_P_). Fatty acid oxidation (FAO) was calculated immediately after ADP addition (i.e., octanoylcarnitine, plus malate and ADP). Trifluoromethoxy carbonyl-cyanide phenylhydrazone (FCCP, two doses of 0.5 μM each) was added to obtain total ETS capacity (uncoupled state). To activate complex IV (CIV) dependent respiration, the first three complexes of the ETS were inhibited by adding rotenone (inhibits complex-I; 0.5 μM), malonic acid and myxothiazol (inhibits complex-II; 5 mM and 0.5 μM, respectively) and antimycin A (inhibits complex-III; 2.5 μM). Sodium ascorbate (2 mM) and N, N, N′, N′-tetramethyl-p-phenylenediamine (TMPD, 0.5 mM) were then added to stimulate CIV supported respiration, which was then inhibited by adding sodium azide (200 mM). ETS excess capacity was calculated using the formula: 1- CI + II_P_/total ETS (1-P/E). Cytochrome c (10 μM) was added to check mitochondrial membrane integrity and data were excluded if respiration increased by >30%. All substrates used were at their saturating concentrations to assess maximal mitochondrial respiratory capacity.

### Placental Lz gene expression analysis

Placental Lz RNA was extracted using the RNeasy Plus Mini Kit (Qiagen, Hilden, UK) and the quantity of RNA obtained was determined using a NanoDrop spectrophotometer (NanoDrop Technologies, Inc., Auburn, AL). A total of 2 μg per sample was reverse transcribed using high-capacity cDNA reverse transcription kit (Applied Biosystems, Foster City, USA) according to the manufacturer’s instructions. Three dilutions of each cDNA sample (1:10, 1:20, and 1:100) were run as a triplicate along with non-template controls in the 7500 Fast RT PCR thermocycler System (Applied Biosystems, UK) for gene expression quantification using gene-specific primer pairs (Table 1) and SYBR Green master-mix (Applied Biosystems, UK). The standard thermal cycling protocol was conducted as follows: 50 °C for 2 min, 95 °C for 10 min and 40 cycles of 95 °C for 95 s and 60 °C for 1 min. Relative expression was calculated using the 2^-ΔΔCt^ method and genes of interest were normalized to the mean expression of three housekeeping genes (*Hprt*, *Tyrosine 3-Monooxygenase/Tryptophan 5-Monooxygenase Activation Protein Zeta (Ywhaz),* and *Ubiquitin C (Ubc)*), which were stable in the placental Lz between the lightest and heaviest fetuses of each sex. Data were then displayed relative to the average mRNA expression value for the heaviest fetus of each sex.

**Table 1:** Primers used for qPCR analysis

Symbol	GenBank ID	Primer sequences (5′ = > 3′) Fw/Rv	Amplicon length (bp)
*Nutrient transporters genes*			
*Slc2a1*	NM_011400.3	Fw: GCTTATGGGCTTCTCCAAACT	123
		Rv: GGTGACACCTCTCCCACATAC	
*Slc2a3*	NM_011401.4	Fw: GA TCGGCTCTTTCCAGTTTG	176
		Rv: CAATCATGCCACCAACAGAG	
*Fatp1*	NM_011977.4	Fw: GGCTCCTGGAGCAGGAACA	65
		Rv: ACGGAAGTCCCAGAAACCAA	
*Fatp3*	NM_011988.3	FW: GAGAACTTGCCACCGTATGC	162
		Rv: GGCCCCTATATCTTGGTCCA	
*Fatp4*	NM_011989.5	Fw: GATTCTCCCTGTTGCTCCTGT	174
		Rv: CCATTGAAGCAAACAGCAGG	
*Fatp6*	NM_001081072.1	FW: AACCAAGTGGTGACATCTCTGC	158
		Rv: TCCATAAAGTAAAGCGGGTCAG	
*Slc38a2*	NM_001355633.1	Fw: TAATCTGAGCAATGCGATTGTGG	129
		Rv: AGATGGACGGAGTATAGCGAAAA	
*Slc38a1*	NM_001166456.1	Fw: CCTTCACAAGTACCAGAGCAC	127
		Rv: GGCCAGCTCAAATAACGATGAT	
*Slc38a4*	NM_001358060	Fw: GCGGGGACAGTATTCAGGAC	102
		Rv: GGAACTTCTGACTTTCGGCAT	
*Slc7a5*	NM_011404.3	Fw: CTGCTGACACCTGTGCCATC	161
		Rv: GGCTTCTTGAATCGGAGCC	
*Slc7a8*	NM_016972.2	Fw: CCAGTGTGTTGGCCATGATC	161
		Rv: TGCAACCGTTACCCCATAGAA	
*Mitochondrial genes*			
*Pgc1*	NM_008904.2	Fw: GCAGTCGCAACATGCTCAAG	83
		Rv: GGGAACCCTTGGGGTCATTT	
*Nrf1*	NM_001164226.1	Fw: AGAAACGGAAACGGCCTCAT	96
		Rv: CATCCAACGTGGCTCTGAGT	
*Nrf2*	NM_010902.4	Fw: ATGGAGCAAGTTTGGCAGGA	96
		Rv: GCTGGGAACAGCGGTAGTAT	
*Tfam*	NM_009360.4	Fw: TCCACAGAACAGCTACCCAA	84
		Rv: CCACAGGGCTGCAATTTTCC	
*Pparγ*	NM_001113418.1	Fw: TGCAGCCTCAGCCAAGTTGAA	77
		Rv: TTCCCGAACTTGACCAGCCA	
*Opa1*	NM_001199177.1	Fw: TGGGCTGCAGAGGATGGT	60
		Rv: CCTGATGTCACGGTGTTGATG	
*Mfn1*	NM_024200.4	Fw: TTGCCACAAGCTGTGTTCGG	148
		Rv: TCTAGGGACCTGAAAGATGGGC	
*Mfn2*	NM_001285920.1	Fw: AGAGGCAGTTTGAGGAGTGC	103
		Rv: ATGATGAGACGAACGGCCTC	
*Drp1*	NM_152816.3	Fw: ATGCCAGCAAGTCCACAGAA	86
		Rv: TGTTCTCGGGCAGACAGTTT	
*Fis1*	NM_025562.3	Fw: CAAAGAGGAACAGCGGGACT	95
		Rv: ACAGCCCTCGCACATACTTT	
*Steroid metabolism and signaling*			
*Star*	NM_011485.5	Fw: TCGCTACGTTCAAGCTGTGT	184
		Rv: GCTTCCAGTTGAGAACCAAGC	
*Cyp11a1*	NM_001346787.1	Fw: GCCCCCGGAGAGCTTG	193
		Rv: TCCCATGCTGAGCCAGA	
*Cyp17a1*	NM_007809.3	Fw: TGGAGGCCACTATCCGAGAA	119
		Rv: CACATGTGTGTCCTTCGGGA	
*Hsd11b1*	NM_008288.2	Fw: GAGGAAGGTCTCCAGAAGGTA	143
		Rv: ATGTCCAGTCCGCCCAT	
*Hsd11b2*	NM_008289.2	Fw: GGCTGGATCGCGTTGTC	132
		Rv: CGTGAAGCCCATGGCAT	
*Esr2*	NM_207707.1	Fw: CCTCGTTCTGGACAGGTCCTC	70
		Rv: CCTTGGGACAGCACTCTTCG	
*Ar*	NM_013476.4	Fw: GGATTCTGTGCAGCCTATTGC	90
		Rv: TCAGGAAAGTCCACGCTCAC	
*Imprinted genes*			
*Igf2P0*	NM_001315488.1	Fw: GAGGAAGCTCTGCTGTTTGG	92
		Rv: CAAAGAGATGAGAAGCACCAAC	
*delta like non-canonical Notch ligand 1 (Dlk1)*	NM_001190705	Fw: GAAAGGACTGCCAGCACAAG	141
		Rv: CACAGAAGTTGCCTGAGAAGC	

Annealing temperature was 60 °C for all genes. *Ar*, Androgen receptor; *Cyp11a1*, Cytochrome P450 Family 11 Subfamily A Member 1; *Cyp17a1*, Cytochrome P450 Family 17 Subfamily A Member 1; *Dlk*, Delta like non-canonical notch ligand 1*; Drp1*, Dynamin 1-like; *Esr2*, Estrogen receptor 2 (beta); *Fatp1*, Solute Carrier Family 27 (Fatty Acid Transporter), Member 1; *Fatp3*, Fatty Acid Transporter, Member 3; *Fatp4*, Fatty Acid Transporter, Member 4; *Fatp6*, Fatty Acid Transporter, Member 6; *Fis1*, Mitochondrial fission factor; *Hsd11b1*, Hydroxysteroid 11-Beta Dehydrogenase 1; *Hsd11b2*, Hydroxysteroid 11-Beta Dehydrogenase 1; *Igf2P0*, Placental specific insulin growth factor 2; *Mfn1*, Mitofusin 1; *Mfn2*, Mitofusin 2; *Nrf1*, Nuclear respiratory factor 1; *Nrf2*, Nuclear respiratory factor 2; *Opa1*, Mitochondrial dynamin like GTPase; *Pgc-1α*, Peroxisome proliferator-activated receptor gamma coactivator 1-alpha; Peroxisome proliferator activated receptor gamma *Pparγ*; *Slc2a1*, Solute Carrier Family 2 (Facilitated Glucose Transporter), Member 1; *Slc2a3*, Solute Carrier Family 2 (Facilitated Glucose Transporter), Member 3; *Slc38a2*, Solute carrier family 38, member 2; *Slc38a1*, Solute carrier family 38, member 1; *Slc38a4*, Solute carrier family 38, member 4; *Slc7a5*, Solute Carrier Family 7 (Amino Acid Transporter Light Chain, L System), Member 5; *Slc7a8*, Solute carrier family 7 (cationic amino acid transporter, y + system), member 8; *Star*, Steroidogenic Acute Regulatory Protein; *Tfam*, Transcription factor A, mitochondria.

**Table 2:** List of primary antibodies used in this study

Primary antibody	Host/isotype	Manufacturer, catalogue number	Dilution
AKT	Rabbit	Cell Signalling, 9272	1/1000
Phospho-AKT (Ser473)	Rabbit	Cell Signalling, 9271	1/1000
AMPK	Rabbit	Cell Signalling, 5832	1/1000
Phospho-AMPK (Thr172)	Rabbit	Cell Signalling, 2535	1/1000
P44/42 MAPK (Erk1/2)	Rabbit	Cell Signalling, 4695	1/1000
Phospo-MAPK-p44/42 (Erk1/2) (Thr202/Tyr204)	Rabbit	Cell Signalling, 4370	1/1000
Total-p38 MAPK	Rabbit	Cell Signalling, 8690	1/1000
Phos-p38 MAPK Thr180/Tyr182	Rabbit	Cell Signalling, 4511	1/1000
OPA1	Rabbit	Cell Signalling, 80 471	1/1000
PGC-1α	Rabbit	Santa Cruz, sc-13 067	1/1000
OXPHOS (ETS complexes)	Mouse	Thermo Fisher, 45–8099	1/250
Citrate synthase	Rabbit	Abcam, ab9660	1/1000
MNF-2	Rabbit	Cell Signalling, 9482	1/1000
HSP60	Rabbit	Abcam, ab46798	1/1000
HSP70	Rabbit	Abcam, ab194360	1/1000
TID1	Rabbit	Genetex, GTX111077	1/1000
CLPP	Rabbit	Abcam, ab124822	1/1000
PPAR-γ	Mouse	Santa Cruz (sc-7273)	1/200

### Placental Lz structural analysis

Morphology of the Lz was assessed by double-labeling placental sections with cytokeratin and lectin antibodies to identify trophoblast and fetal capillaries, respectively. Details about the staining protocol have been described in detail elsewhere [29]. Stained sections were then scanned using a NanoZoomer 2.0-RS Digital Pathology System (NDP Scan Hamamatsu, Japan) and stereological analysis of the Lz was performed as described previously [29].

### Statistical analysis

Statistical analyses were performed using GraphPad Prism version 8 (GraphPad, La Jolla, CA, USA) and SAS/STAT (Statistical System Institute Inc. Cary, NC, USA). To check whether each data set was normally distributed, Shapiro–Wilk tests were performed and identified that all displayed a normal Gaussian distribution. To compare conceptus biometry between males and females across different litters, linear mixed model analyses were performed (which considers each fetus as a repeat in the litter) and used the number of viable pups per litter as a covariate. Our primary objective was to assess differences between the lightest and heaviest fetuses for each sex within the litter. To achieve this, differences in placental structure, mitochondrial respiration, and gene and protein expression between the heaviest and the lightest fetuses of each sex within the litter were assessed by paired *t*-tests. Retrospective comparisons between the lightest females and lightest males and heaviest females and heaviest males within the litter were also performed by paired *t-*tests. Relationships between data were undertaken using Pearson’s correlation coefficient (*r*). Values are expressed as individual data points and/or mean ± SEM and *P*-values < 0.05 were considered statistically significant. The number of samples per group for each analysis is shown in each figure and described in the legends of figures and footnotes of tables.

## Results

### Conceptus biometry for all females versus males within the litter

**Figure 1:**
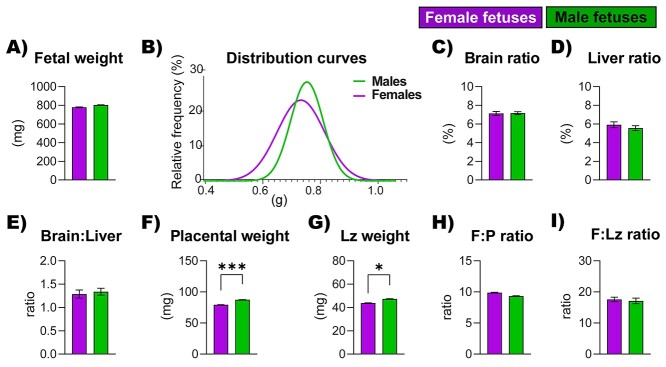
Conceptus biometrical data from female and male fetuses at GD 18. (A) Fetal weight, (B) fetal weight distribution curves, (C) brain, (D) liver weights as a proportion of fetal weight, (E) brain to liver weight ratio, (F) placental weight, (G) Lz weight, (H) placental efficiency (F:P ratio; determined as the ratio of fetal weight to placental weight), and (I) Lz efficiency (F : Lz ratio). Data are from *n* = 13 litters and are displayed as mean ± SEM. Aside from (B), data were analyzed by Linear mixed model with litter size as a covariate; ^*^P < 0.05, ^***^P < 0.001.

Considering all fetuses together, the weight of female and male fetuses did not significantly vary at GD 18 (the term on ~day 20; Figure 1A). The distribution of fetal weights for females and males also did not differ (Figure 1B). Moreover, there were no differences in fetal brain and liver weights (*P* = 0.06, relative to body weight), including the fetal brain to the liver ratio (Figure 1E). However, both placental and Lz weights were lower in females when compared with male fetuses (*P* < 0.001 and *P* = 0.012 respectively; Figure 1F and G). Placental and Lz efficiency, calculated as the ratio of fetal weight to placental and Lz weight were not different between females and males (Figure 1H and I).

**Figure 2:**
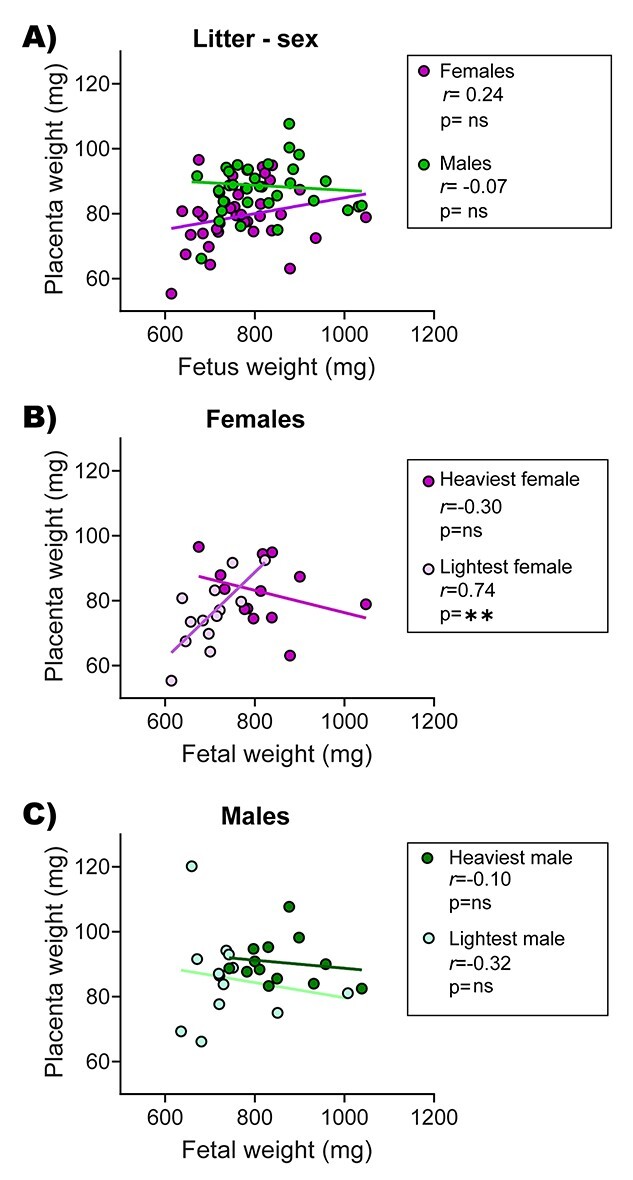
Relationships between placental and fetal weights (A) for each fetal sex when using all data obtained for each litter, (B) using data obtained for just the lightest and heaviest female fetuses in the litter, and (C) using data obtained for just the lightest and heaviest male fetuses in the litter on GD 18. Data were analyzed using Pearson’s correlation coefficient and *r* values are shown in each corresponding figure (^**^P < 0.01). In A, data are from 35 females and 36 males from *n* = 13 litters. In B and C, data are from 12 lightest and 13 heaviest fetuses and respective placentas per sex from *n* = 13 litters. ns = not significant.

Placental weight was not significantly correlated with fetal weight when analyzing all conceptuses within the litter collectively (*n* = 71; *r* = 0.17; *P* = 0.15). Similarly, when data were separated into females and males, again no correlation was detected (Figure 2A, females: *n* = 35; *r* = 0.24; *P* = 0.15, males: *n* = 36; *r* = −0.07 *P* = 0.64). However, when data were segregated to only assess the heaviest and lightest fetuses per sex within the litter, a positive correlation between placental and fetal weight was found for the lightest females (Figure 2B, *r* = 0.74; *P* = 0.005), but not for the lightest males (Figure 2C, *r* = 0.32; *P* = 0.31) or the heaviest fetuses of either sex. These data suggest that the placenta may be supporting the growth of the female and male fetuses in different ways within the litter.

### Conceptus biometry and placental Lz morphology for the lightest versus the heaviest fetuses of each sex

**Figure 3:**
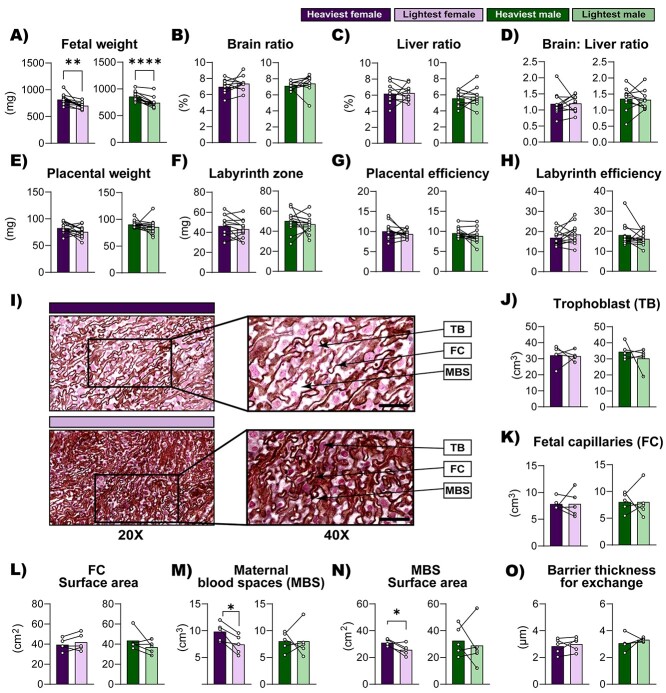
Conceptus biometry and Lz structure of the lightest versus the heaviest fetuses of each fetal sex within the litter on GD 18. (A) Fetal weight, (B) brain, (C) liver weights as a proportion of fetal weight, (D) brain weight to liver weight ratio, (E) placenta weight, (F) Lz weight, (G) placental efficiency, (H) labyrinth efficiency (determined as the ratio of fetal weight to labyrinth weight) and (I) representative images at low (20X) and high (40X) magnification of placental labyrinth histology of female fetuses. Scale bars represent 50 μm. (J) Trophoblast volume, (K) fetal capillaries volume, (L) fetal capillaries surface area, (M) maternal blood spaces volume, (N) maternal blood spaces surface area, and (O) barrier thickness. For conceptus biometry analysis, 13 lightest and 13 heaviest fetuses and respective placentas per sex were used. For stereological analysis, 5 lightest and 5 heaviest fetuses and respective placentas per sex were used. Data are displayed as individual data points with bars representing the mean value and lines connecting siblings from the same litter. Data were analyzed for each sex separately using paired *t* test; ^*^P < 0.05 ^**^P < 0.01 and ^****^P < 0.0001. Abbreviations: TB (trophoblasts); FC (fetal capillaries); MBS (maternal blood spaces).

To understand why there are differences in the relationship between placental weight and fetal weight, the lightest and heaviest fetuses from each litter were selected and conceptus biometry was compared for each sex separately (Figure 3). As expected, fetal weight was lower for the lightest compared to the heaviest for each fetal sex in the litter (Figure 3A, females; *P* = 0.002; males; *P* < 0.0001), and the mean weight difference between them were similar for females and males (14.1% and 13.6% less than heaviest, respectively). Fetal brain and liver weights as a proportion of body weight did not vary, which suggests that the lightest fetuses are symmetrically smaller when compared to the heaviest fetuses (Figure 3B–D). Moreover, placental weight, Lz weight, and placenta and Lz efficiency did not vary between the lightest and the heaviest fetuses within the litter, regardless of fetal sex (Figure 3E–H).

Stereological analysis of the placental Lz zone revealed that there were no differences in trophoblast and fetal capillary volumes (Figure 3I–K). However, there were fewer maternal blood spaces in the placental Lz of the lightest females, compared to the heaviest females, and this difference was not found for the males (Figure 3M). Similarly, maternal blood space surface area was lower in the lightest, compared to heaviest female fetuses, an effect not observed for the male fetuses (Figure 3N). The surface area of the fetal capillaries (Figure 3L) and barrier thickness (Figure 3O) of the Lz did not vary between the lightest and the heaviest fetuses, for either females or males.

### Mitochondria respiratory capacity of the placental Lz for the lightest versus the heaviest fetuses of each sex

**Figure 4:**
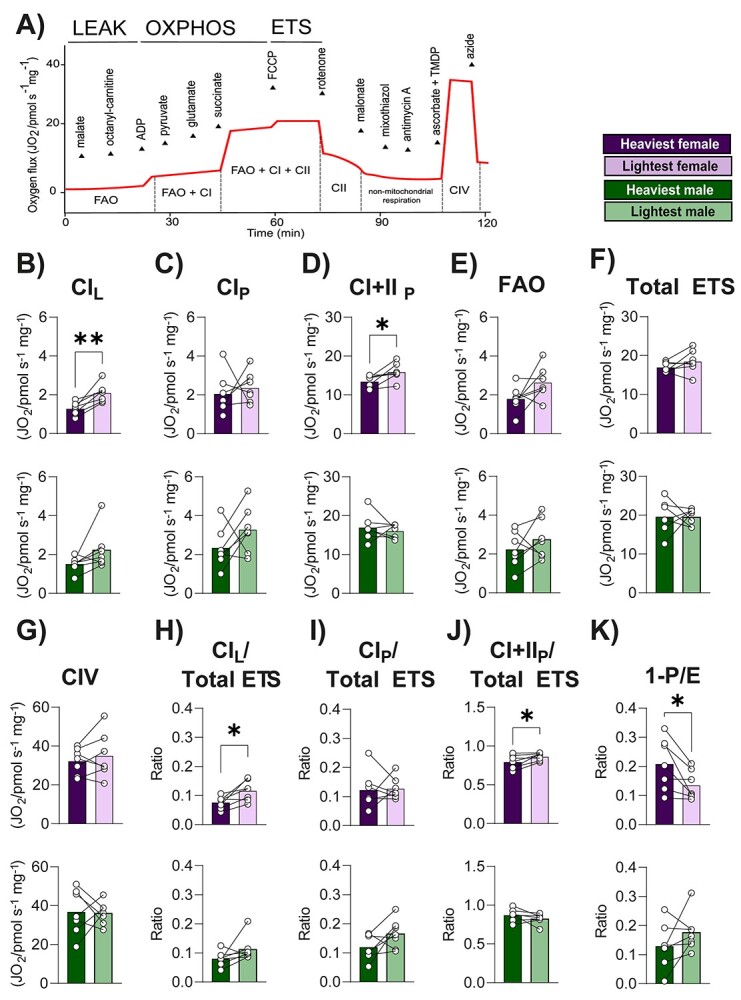
Mitochondrial respiration rates in the Lz support the lightest and heaviest fetuses of each sex within the litter on GD 18. (A) Representative experimental trace, (B) CI_L_: CI_Leak_, (C) CI_P_: CI_Oxphos_, (D) CI + II_P_: CI + CII_Oxphos_, (E) FAO, (F) Total ETS, (G) CIV, (H) C_L_/Total ETS, (I) C_P_/Total ETS, (J) CI + II_P_/Total ETS and (K) 1-P/E. The sample size was 7 lightest and 7 heaviest fetuses per sex from *n* = 7 litters. Data are displayed as individual data points with bars representing the mean value and lines connecting siblings from the same litter. Data were analyzed for each sex separately using paired *t* test; ^*^P < 0.05 and ^**^P < 0.01.

To investigate whether structural changes in the Lz zone between the lightest and heaviest fetuses may be related to mitochondrial functional alterations, high-resolution respirometry was performed on each sex separately (Figure 4A). Oxygen flux rate analysis revealed that in LEAK state, mitochondrial CI-related oxygen consumption was ~60% greater for the placental Lz of lightest compared to the heaviest females, but no effect was seen for the males (Figure 4B, *P* = 0.003). While CI_P_ state was not different between the lightest and heaviest fetuses of either sex (Figure 4C), after adding succinate, Lz CI + CII oxygen consumption rate was ~44% greater in the lightest compared to the heaviest females within the litter (*P* = 0.01); a difference that was not observed for the males (Figure 4D). FAO, total ETS capacity, and CIV-associated oxygen consumption rates by the placental Lz were not different between lightest and heaviest fetuses for either fetal sex (Figure 4E–G). When oxygen consumption rates for CI in LEAK state and CI + II in OXPHOS state were corrected to total ETS oxygen flux to provide a qualitative indication of changes in mitochondrial function per mitochondrial unit, these values were also increased in only the lightest compared to heaviest females (not different for the lightest compared to heaviest males) (Figure 4H, *P* = 0.02; 3I and 3 J, *P* = 0.03). In addition, calculation of 1-P/E indicated that ETS excess capacity was lower in the lightest females compared to the heaviest (*P* = 0.03), again a difference not identified for the males (Figure 4K).

### Expression of mitochondrial ETS components, dynamic genes, and regulatory proteins in the placental Lz for the lightest versus the heaviest fetuses of each sex

**Figure 5:**
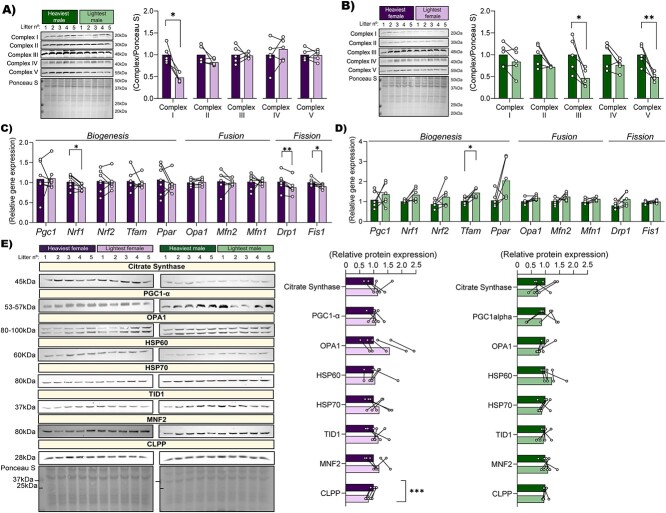
Protein abundance of electron transport chain complexes for each fetal sex (A and B, males and females, respectively). Relative mRNA expression of key mitochondria dynamics genes associated with biogenesis, fusion, and fission processes on females (C) and males (D) and key mitochondrial regulatory proteins; (E) citrate synthase, PGC1α, MNF2, OPA1, HSP60, HSP70, TID1, and CLPP in the placental Lz supporting the lightest and heaviest fetuses of each sex within the litter on GD 18. Images from each antibody and representative Ponceau staining are included. For western blot results, the sample size is 5 lightest and 5 heaviest fetuses per sex from *n* = 5 litters. For qPCR results, the sample size is 7 lightest and 7 heaviest fetuses per sex from *n* = 7 litters. Data are displayed as individual data points with bars representing the mean value and lines connecting siblings from the same litter. Data are displayed relative to the value for the heaviest fetus per sex. Data were analyzed for each sex separately by paired *t* test; ^*^P < 0.05, ^**^P < 0.01, ^***^P < 0.001.

To gain further information about the differences in the placental mitochondrial respiratory capacity, western blotting and qPCR was performed to determine the expression of ETS complex proteins (CIV), biogenesis, fusion and fission genes, and additional mitochondrial regulatory proteins in the placental Lz of the lightest and heaviest fetuses for both sexes (Figure 5). These analyses revealed that CI protein abundance was lower in the lightest compared to heaviest females (Figure 5A), meanwhile, CIII and CV proteins were lower only in the lightest compared to the heaviest males (Figure 5B). In addition, the expression of mitochondria biogenesis gene, nuclear respiratory factor 1 (*Nrf1*), and mitochondrial fission genes, dynamin-related protein (*Drp1)* and Mitochondrial fission factor (*Fis1*), was lower in the Lz of the lightest females, when compared with the heaviest females (Figure 5C). Whereas the expression of transcription factor A, mitochondria (*Tfam*), a mitochondria biogenesis transcription factor gene, was greater in the Lz of the lightest males versus the heaviest males (Figure 5D). Mitochondrial content, informed by citrate synthase protein abundance, did not vary in the Lz between the lightest and heaviest fetuses within the litter, regardless of fetal sex (Figure 5E). In addition, abundance of mitochondrial biogenesis (PGC-1α), fusion (MNF2 and OPA1), heat shock (HSP60, HSP70), and chaperone (TID1, tumorous imaginal disc) proteins did not differ in the Lz between the lightest and heaviest fetuses within the litter, in either sex (Figure 5E). However, protein abundance of caseinolytic mitochondrial matrix peptidase proteolytic subunit (CLPP), a key protease involved in mitochondrial protein clearance and a marker of the mitochondrial unfolded protein response, was lower in the lightest females compared with the heaviest females; an effect not seen for males (Figure 5E).

### Expression of transport genes and steroid metabolism and signaling genes in the placental Lz for the lightest versus the heaviest fetuses of each sex

**Figure 6:**
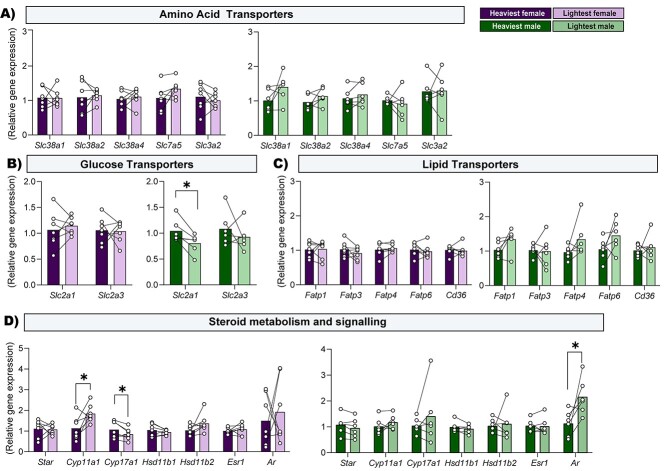
Relative mRNA expression of the amino acid (A), glucose (B), lipid (C) transporters, and steroid hormone metabolism and signaling-related genes (D) in the placental Lz supporting the lightest and heaviest fetuses of each fetal sex within the litter on GD 18. Data are from 7 lightest and 7 heaviest fetuses per sex from *n* = 7 litters. Relative expression was calculated using the 2^-ΔΔCt^ method and genes of interest were normalized to the mean expression of 3 housekeeping genes (*Hprt, Ywhaz* and *Ubc*). Data are displayed as individual data points with bars representing the mean value and lines connecting siblings from the same litter. Data are displayed relative to the value for the heaviest fetus per sex. Data were analyzed for each sex separately using paired *t* test; ^*^P < 0.05.

Since the energy provided by mitochondria helps to fuel placenta transport and endocrine function, we evaluated whether variations found in mitochondria functional capacity (respiratory function, gene, and protein regulators) are associated with the expression of nutrient transporter and steroidogenic genes between the lightest and heaviest for each sex within the litter. In particular, the mRNA expression of key transporters for glucose solute carrier family 2 facilitated glucose transporter member 1 and 3 (*Slc2a1* and *Slc2a3*), amino acid (solute carrier family 38, member 1 and 2 (*Slc38a1*, *Slc38a2*), solute carrier family 38, member 4 (*Slc38a4*)*,* Solute carrier family 7, member 5 (*Slc7a5*) and *Solute Carrier Family 3 Member 2 (Slc3a2)*) and lipids fatty acid transporter member 1–6 (*Fatp1, Fatp3, Fatp4, Fatp6,* and *CD36 molecule (Cd36)*) were quantified in the placental Lz zone by RT-qPCR (Figure 6). We also evaluated the expression of genes involved in steroid hormone production (*Star,* cytochrome P450 family 11 subfamily A member 1 (*Cyp11a1*), and cytochrome P450 family 17 subfamily A member 1 (*Cyp17a1*), glucocorticoid metabolism (*Hsd11b1* and *Hsd11b2*), and steroid hormone signaling (estrogen receptor beta (*Esr2*) and androgen receptor (*Ar*)) in the placental Lz using qPCR. These analyses showed that the expression of *Slc2a1* mRNA was ~20% lower for the lightest compared to the heaviest males (Figure 6B, *P* = 0.021), however, this difference was not observed for the lightest versus the heaviest females. In addition, no differences were found between the lightest and the heaviest fetuses within the litter for any of the other nutrient transporter genes quantified in either fetal sex (Figure 6A–C). The gene expression of *Cyp11a1* was ~63% greater (*P* = 0.038), while *Cyp17a1* ~ 20% lower (*P* = 0.035) in the lightest compared to the heaviest female fetus, with no differences in these steroidogenic genes detected in the males (Figure 6D). Whereas the mRNA expression of the (*Ar*) a steroid-hormone activated transcription factor was ~91% greater (*P* = 0.046) in the lightest compared to the heaviest males only (Figure 6D). The mRNA expression of *Esr2*, the 11β-hydroxysteroid dehydrogenase isozymes 1 and 2 (*Hsd11b1* and *Hsd11b2*), and *Star* in the Lz were not different between the lightest compared to the heaviest fetuses in either sex.

### Abundance of key growth and metabolic proteins in the placental Lz for the lightest versus the heaviest fetuses of each sex

**Figure 7:**
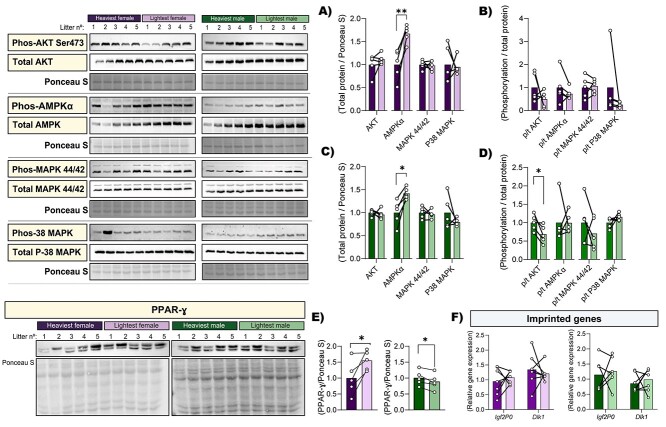
Protein abundance of key growth and metabolic signaling proteins in the placental Lz supporting the lightest and heaviest fetuses of each sex within the litter on GD 18. (A and C) Total AKT, AMPKα, MAPK 44/42 and P38 MAPK protein levels, and (B and D) AKT, AMPKα, MAPK 44/42 and P38 MAPK phosphorylation levels as a ratio to total protein in the heaviest versus the lightest fetuses for females (A and B) and males (C and D). Total protein abundance for PPARγ (E). Relative mRNA expression of imprinted genes *Igf2P0* and *Dlk1* (F). Representative images for data including Ponceau staining for (A)–(E) are included. Protein abundance and phosphorylation levels were normalized to Ponceau staining and total protein abundance, respectively. Relative expression was calculated using the 2-ΔΔCt method and genes of interest were normalized to the mean expression of 3 housekeeping genes (*Hprt*, *Ywhaz,* and *Ubc*). Data are 5 lightest and 5 heaviest fetuses per sex from *n* = 5 litters. Data are displayed as individual data points with bar representing the mean value and the lines connecting siblings from the same litter. Data are displayed relative to the value for the heaviest fetus per sex. Data were analyzed for each sex separately by paired *t* test; ^*^P < 0.05.

To provide information related to the differences in placental morphology and mitochondrial function between lightest and heaviest fetuses, the abundance of key growth and metabolic signaling proteins, namely protein kinase B (AKT), AMPKα (5’-AMP-activated protein kinase catalytic subunit alpha-1), p44/42 mitogen-activated protein kinase (MAP) (ERK1/ERK2), p38 MAPK and peroxisome proliferator-activated receptor-gamma (PPARγ) were evaluated by western blotting (Figure 7). The abundance of total AMPKα protein was greater in the lightest compared to the heaviest fetuses for both females and males (67% and 41%, Figure 7A and C, *P* = 0.005 and *P* = 0.01, respectively), however, this was not related to a significant change in activation of AMPKα (abundance of phosphorylated AMPKα normalized to total AMPKα protein, Figure 7B and D). While the total abundance of AKT protein did not vary between the lightest and the heaviest fetuses, activated AKT (phosphorylated to total AKT protein) was ~32% lower in the Lz zone supporting the lightest compared to the heaviest males (*P* = 0.032), but no difference was found for the females (Figure 7B and D). The abundance and activation of p44/42 MAPK and p38 MAPK proteins were not different between the lightest and the heaviest fetuses, irrespective of fetal sex (Figure 7A and D). Interestingly, the abundance of PPARγ, an important transcription factor involved in mitochondrial metabolism and lipid synthesis, was greater in the lightest female compared to the heaviest female, whereas PPARγ protein was lower in the lightest males when compared to the heaviest males from the litter (Figure 7E, *P* = 0.04 and *P* < 0.05, respectively). In addition, we evaluated the expression of imprinted genes Placental specific insulin growth factor 2 (*Igf2P0*) and *Dlk1,* which are involved in feto-placental growth and allocation of maternal resources [30, 31], and found no differences between the heaviest and the lightest of each sex (Figure 7F).

### Retrospective comparisons of the effect of sex on feto-placental growth of the lightest and heaviest fetuses

To gain further insight into the intra-litter differences in placental phenotype, retrospective comparisons between the lightest females and lightest males and heaviest females and heaviest males within the litter were performed (Table 3). These data showed that the heaviest males were ~ 5% heavier than the heaviest female fetuses within the litter (*P* < 0.05). The placenta of the heaviest males in the litter was also greater by ~13% when compared to the heaviest females (*P* = 0.04), with a tendency for this to also vary with sex for the lightest female littermates (*P* = 0.054). The placental expression of glucose (*Slc2a1*: –37%, *P* = 0.003) and lipid (*Fatp1*: +17%, tendency *P* = 0.07) transporter genes were also differentially expressed between the lightest (but not heaviest) male and female fetuses of the litter. Placental respirometry rates associated with CI_Oxphos_ (+39%, tendency *P* = 0.05) and with CI + CII_Oxphos_/Total ETS (+30%, tendency *P* = 0.08) together with biogenesis (*Nrf1*: +37%, *P* = 0.03; *Tfam*: +25%, tendency *P* = 0.06) and dynamic (*Opa1*: +13%, tendency *P* = 0.06, *Mfn1*: +11%, *P* = 0.05) genes were all greater in the lightest males compared to the lightest females. Meanwhile, biogenesis genes *Pparγ* (−38%, *P* = 0.03) and *Tfam* (−19%, tendency *P* = 0.07) were decreased in the heaviest males compared to females. Finally, the expression of the steroidogenic gene *Cyp11a1* was lower (−38%, *P* = 0.02) in the lightest males compared to females and in heaviest fetuses, gene expression of *Cyp17a1* (−41%, *P* = 0.01) was decreased in males compared to females. There was also no effect of fetal sex in the placental Lz morphology of the lightest and heaviest fetuses.

**Table 3:** Comparisons between fetal sexes for the lightest and heaviest fetuses

	Lightest			Heaviest		
	Female	Male	*P*-value	Female	Male	*P*-value
*Conceptus biometry*						
Fetal weight (mg)	702.13 ± 15.91	740.61 ± 26.68	NS	817.33 ± 25.85	857.60 ± 22.66	*P* < 0.05
Placenta weight (mg)	75.75 ± 2.88	85.74 ± 3.77	*P* = 0.05	82.62 ± 2.67	90.54 ± 1.95	*P* < 0.05
Lz weight (mg)	43.18 ± 2.50	46.97 ± 2.34	NS	46.29 ± 2.90	50.15 ± 2.97	NS
Liver weight (mg)	43.08 ± 2.19	42.55 ± 2.22	NS	49.61 ± 3.70	47.03 ± 2.41	NS
Brain weight (mg)	50.48 ± 1.82	55.57 ± 3.46	NS	55.59 ± 2.60	60.25 ± 1.88	NS
Brain/liver ratio	1.20 ± 0.08	1.35 ± 0.12	NS	1.19 ± 0.12	1.32 ± 0.09	NS
Placenta efficiency	9.37 ± 0.26	8.84 ± 0.49	NS	10.07 ± 0.54	9.53 ± 0.36	NS
Labyrinth zone efficiency	16.90 ± 1.01	16.19 ± 0.87	NS	18.59 ± 1.36	18.05 ± 1.53	NS
*Labyrinth zone structure*						
Trophoblast (mm^3^)	31.6 ± 1.4	30.3 ± 2.9	NS	32.1 ± 2.7	34.4 ± 2.2	NS
Fetal capillaries (mm^3^)	7.9 ± 1.1	8.0 ± 1.3	NS	7.8 ± 0.5	8.0 ± 0.8	NS
Maternal blood spaces (mm^3^)	7.4 ± 0.8	9.3 ± 1.6	NS	9.8 ± 0.8	11.0 ± 1.7	NS
Fetal capillaries (cm^2^)	41.9 ± 3.8	36.9 ± 2.9	NS	39.4 ± 2.7	43.5 ± 4.5	NS
Maternal blood spaces (cm^2^)	25.7 ± 1.9	29.0 ± 7.5	NS	31.0 ± 1.1	32.6 ± 5.0	NS
Barrier thickness (μm)	3.0 ± 0.2	3.3 ± 0.1	NS	2.8 ± 0.3	3.1 ± 0.3	NS
*Growth/nutrient signaling*			ND			ND
*Transporter gene expression* (relative expression, arbitrary units)						
* Slc2a1*	1.21 ± 0.07	0.79 ± 0.07	*P* < 0.05	1.07 ± 0.14	1.03 ± 0.09	NS
* Slc2a3*	1.06 ± 0.08	0.93 ± 0.11	NS	1.06 ± 0.10	1.09 ± 0.14	NS
* Fatp1*	1.04 ± 0.10	1.22 ± 0.12	*P* = 0.07	1.02 ± 0.08	0.94 ± 0.09	NS
* Fatp3*	1.03 ± 0.09	1.03 ± 0.19	NS	1.02 ± 0.08	1.04 ± 0.07	NS
* Fatp4*	1.01 ± 0.04	1.27 ± 0.20	NS	1.02 ± 0.07	0.96 ± 0.08	NS
* Fatp6*	1.02 ± 0.08	1.21 ± 0.15	NS	1.02 ± 0.08	0.80 ± 0.10	NS
* Cd36*	1.01 ± 0.06	1.09 ± 0.15	NS	1.01 ± 0.05	0.97 ± 0.07	NS
* Slc38a1*	1.03 ± 0.09	1.22 ± 0.14	NS	1.08 ± 0.11	0.91 ± 0.12	NS
* Slc38a2*	1.02 ± 0.06	0.96 ± 0.08	NS	1.09 ± 0.17	0.92 ± 0.08	NS
* Slc38a4*	1.04 ± 0.09	1.23 ± 0.14	NS	1.04 ± 0.08	1.19 ± 0.13	NS
* Slc7a5*	1.02 ± 0.07	0.81 ± 0.14	NS	1.07 ± 0.14	1.18 ± 0.06	NS
* Slc3a2*	1.04 ± 0.09	1.10 ± 0.19	NS	1.11 ± 0.15	0.96 ± 0.13	NS
*Mitochondria respiratory function* (*J*O_2_, P (pmol s^−1^ mg ^−1^))						
Complex I LEAK	2.09 ± 0.18	2.24 ± 0.40	NS	1.27 ± 0.12	1.51 ± 0.14	NS
Complex I OXPHOS	2.37 ± 0.30	3.28 ± 0.45	*P* = 0.05	2.05 ± 0.40	2.34 ± 0.40	NS
Complex I + II OXPHOS	15.92 ± 0.84	16.04 ± 0.66	NS	13.40 ± 0.60	16.93 ± 1.31	*P* = 0.08
FAO	2.64 ± 0.31	2.75 ± 0.38	NS	1.79 ± 0.25	2.23 ± 0.35	NS
Total ETS	18.48 ± 1.09	19.53 ± 0.78	NS	16.95 ± 0.49	19.61 ± 1.60	NS
CIV	34.94 ± 4.42	36.07 ± 2.21	NS	32.17 ± 2.55	36.79 ± 4.44	NS
Complex I LEAK/ETS	0.12 ± 0.01	0.11 ± 0.02	NS	0.08 ± 0.01	0.08 ± 0.01	NS
Complex I OXPHOS/ETS	0.12 ± 0.01	0.17 ± 0.02	*P* = 0.06	0.12 ± 0.02	0.12 ± 0.02	NS
Complex I + II OXPHOS/ETS	0.87 ± 0.02	0.82 ± 0.03	NS	0.79 ± 0.03	0.87 ± 0.03	NS
1-P/E	0.14 ± 0.02	0.18 ± 0.03	NS	0.21 ± 0.03	0.13 ± 0.03	NS
*ETS complex proteins*			ND			ND
*Mitochondria-related gene expression* (relative expression, arbitrary units)						
* Pgc1α*	1.03 ± 0.11	1.28 ± 0.21	NS	1.09 ± 0.15	1.09 ± 0.17	NS
* Nrf1*	1.01 ± 0.03	1.38 ± 0.14	*P* < 0.05	1.02 ± 0.06	0.90 ± 0.03	NS
* Nrf2*	1.02 ± 0.08	1.23 ± 0.22	NS	1.04 ± 0.10	0.87 ± 0.08	NS
*Tfam*	1.02 ± 0.07	1.28 ± 0.08	*P* = 0.06	1.04 ± 0.09	0.84 ± 0.07	*P* = 0.07
*Pparγ*	1.03 ± 0.09	1.38 ± 0.28	NS	1.07 ± 0.14	0.66 ± 0.10	*P* < 0.05
*Opa1*	1.00 ± 0.03	1.13 ± 0.05	*P* = 0.06	1.00 ± 0.03	0.97 ± 0.06	NS
*Mfn2*	1.00 ± 0.03	1.13 ± 0.07	NS	1.03 ± 0.09	0.92 ± 0.07	NS
*Mfn1*	1.01 ± 0.03	1.12 ± 0.04	*P* = 0.05	1.02 ± 0.07	0.99 ± 0.06	NS
*Drp1*	1.03 ± 0.08	1.30 ± 0.15	NS	1.03 ± 0.09	0.82 ± 0.08	NS
*Fis1*	1.00 ± 0.02	1.11 ± 0.04	NS	1.00 ± 0.03	0.94 ± 0.03	NS
*Mitochondria-related proteins*			ND			ND
*Steroid handling genes* (relative expression, arbitrary units)						
*Star*	1.05 ± 0.09	1.22 ± 0.20	NS	1.12 ± 0.16	1.38 ± 0.20	NS
*Cyp11a1*	1.03 ± 0.09	0.78 ± 0.08	*P* < 0.05	1.13 ± 0.22	1.15 ± 0.15	NS
*Cyp17a1*	1.05 ± 0.13	1.05 ± 0.34	NS	1.06 ± 0.15	0.63 ± 0.07	*P* < 0.05
*Hsd11b1*	1.01 ± 0.05	0.98 ± 0.07	NS	1.05 ± 0.11	0.99 ± 0.08	NS
*Hsd11b2*	1.05 ± 0.13	0.80 ± 0.18	NS	1.03 ± 0.09	0.97 ± 0.11	NS
*Esr1*	1.04 ± 0.10	1.04 ± 0.14	NS	1.00 ± 0.06	1.10 ± 0.11	NS
*Ar*	1.40 ± 0.43	0.88 ± 0.12	NS	1.50 ± 0.46	0.63 ± 0.11	NS
*Imprinted genes* (relative expression, arbitrary units)						
*Igf2P0*	1.08 ± 0.22	1.26 ± 0.20	NS	0.95 ± 0.46	1.15 ± 0.22	NS
*Dlk1*	1.21 ± 0.27	0.99 ± 0.15	NS	1.38 ± 0.56	0.86 ± 0.11	NS

Data from qPCR and high-resolution respirometry analyses are from 7 lightest and 7 heaviest fetuses per sex from *n* = 7 litters. Data from fetal biometrical assessments results are from 13 lightest and 13 heaviest fetuses per sex from *n* = 13 litters. Data from placenta stereology are from 5 lightest and 5 heaviest fetuses per sex from *n* = 5 litters. Data are displayed as mean ± SEM and analyzed for the effect of sex in each fetal weight category by paired *t* test ^*^P < 0.05. ND, not determined; NS, not significant. ^*^P < 0.05.

## Discussion

**Figure 8:**
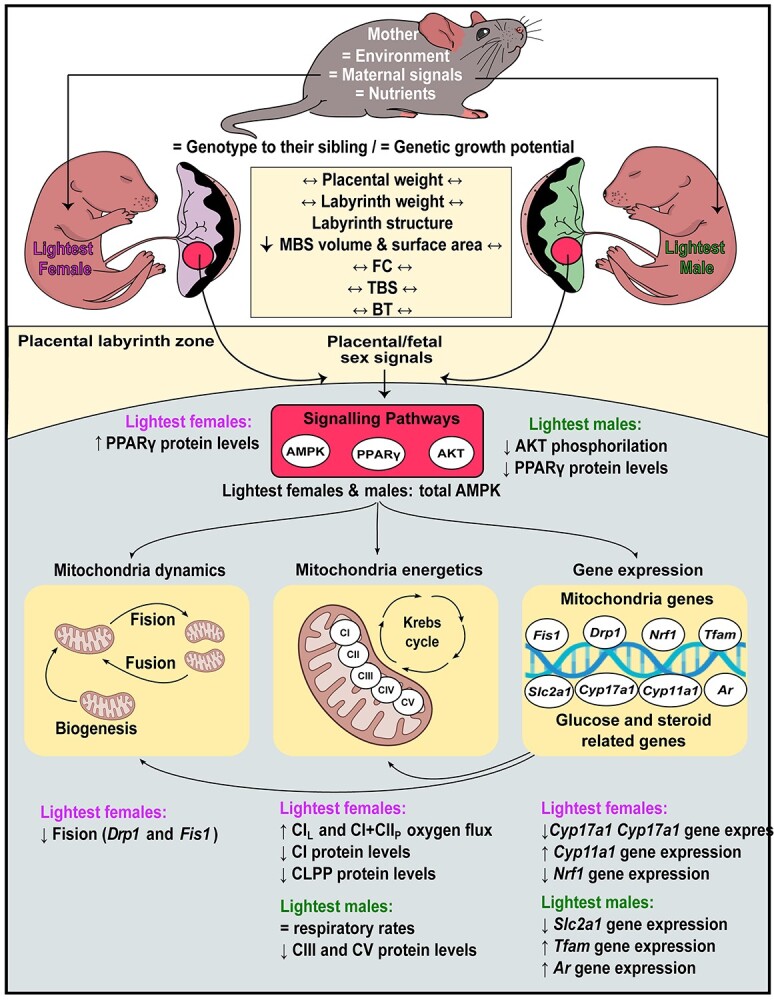
Summary figure representing the alterations in placental phenotype of the lightest versus heaviest fetuses of each sex in normal pregnant mice at GD 18. Fetuses within the litter are exposed to the same maternal endocrine and nutritional environment, yet differences in the weight of fetuses can be observed. A comprehensive analysis of the lightest and heaviest fetus from the litter revealed significant structural, functional, mitochondrial, and molecular differences in the placental Lz, which in turn, differed for each fetal sex. We speculate that in part, these sex-dependent differences in placental phenotype between the lightest and heaviest fetuses of the litter are due to variations in the fetal endocrine and metabolic environment which operate via key signaling pathways (AMPK, AKT, and PPARγ) in the placental Lz . Further work is required to provide a mechanistic explanation for differences seen in placental phenotype with fetal weight for the two sexes and to understand their physiological relevance in suboptimal gestational environments.

In line with other studies, this study in mice showed that feto-placental weight is on average, greater for males compared to females. Furthermore, the relationship between fetal weight and placental weight varied for each fetal sex within the litter. The principal aim of this study was therefore to understand how fetal weight differences in normal physiological mouse pregnancies relate to placental function in the two sexes separately. We showed that placental mitochondrial functional capacity does indeed alter with respect to natural differences in the weight of females and male fetuses within the litter. The placental Lz of the lightest female and male fetuses showed the altered abundance of ETS complex proteins and mitochondrial biogenesis genes when compared with their respective heaviest counterparts, however, the specific nature of these changes differed in females and males. Moreover, the morphology, respiratory capacity, mitochondrial fission, and abundance of misfolded protein regulators of the placental Lz differed between the lightest and heaviest females, but not males. Furthermore, the level of nutrient (glucose) transporter genes varied between the lightest and heaviest males, but not females, whereas the ability to produce steroid hormones (informed by expression of steroidogenic enzyme genes) differed only between the lightest and heaviest females within the litter. There were also changes in the expression of hormone-responsive genes, and growth and metabolic signaling proteins in the placental Lz between the lightest and heaviest fetuses of each sex. Despite dissimilarities in the changes seen for each sex, the average weight difference between the lightest and heaviest fetuses was similar for both sexes. Together, these data suggest that in normal mouse pregnancy, placental structure, function, and mitochondrial phenotype appear to respond differentially to the genetically determined growth demands of the female and the male fetus (Figure 8).

### Fetal and placenta growth

In our study, placental efficiency, and other fetal biometry parameters (fractional liver and brain weights) were not different between the lightest and heaviest fetuses of each sex. This is relevant since placental efficiency indicates the capacity of the placenta to support fetal growth and alterations in this measure, as well as the symmetry of fetal body growth, enhances the risk for chronic diseases in later life through developmental programming [13]. Similarities in body proportionality between lightest and heaviest fetuses likely relate to the adaptative properties of their placentas in these normal, healthy pregnancies. Interestingly, previous studies exploring the implications of natural intra-litter variability of placental weight, rather than fetal weight in mice, have found morphological differences between the lightest and the heaviest placentas, which included a greater Lz volume and an increased surface area for exchange [3]. Functional adaptations were also found, with a greater rate of amino acid transfer and enhanced expression of sodium-dependent neutral amino acid transporter-2 (*Slc38a2*) by the lightest versus the heaviest placentas [3]. Indeed, recent studies in mice have demonstrated that placenta-specific knockdown of the *Slc38a2* gene causes FGR [32]. Similarly, calcium transfer across the lightest placenta was higher than the heaviest placentas within the litter, resulting in similar calcium accretion levels in the fetus [6]. Variations in placental structure and transport within the litter were related to an increase in placental efficiency in both of these previous studies [3, 6]. However, in our study, placentas sustaining the lightest or the heaviest fetuses were not necessarily the lightest or the heaviest placentas within the litter. In addition, a key strength of our study is that the lightest and heaviest fetuses of each sex were analyzed. Segregating the data by sex identified that there was a positive correlation between placental and fetal weight for only the lightest females in the litter. These data indicate that there may be differences in the way in which the placenta may be supporting the growth of the female and male fetuses within the litter. This is consistent with other work in humans suggesting that the relationship between placental weight and birth weight differs statistically between females and males and may reflect sexual dimorphism in placental reserve capacity and prioritization of somatic growth [33].

While there was no difference in placental fetal capillaries, trophoblast volume or barrier thickness between the lightest and heaviest of either fetal sex, maternal blood space volume, and placenta surface area were lower in the lightest compared to the heaviest female fetuses. These differences in placental structure suggest mal-perfusion of the placenta of the lightest females and would be expected to decrease the delivery of nutrients and oxygen to the fetus, and could explain the weight discrepancy with the heaviest females in the litter. Indeed, previous studies in pigs have shown that compared to placentas supporting fetuses weighing closest to the litter mean, placentas supplying the lightest fetuses within the litter have impaired angiogenesis [34]. Moreover, work in rats has suggested that an angiogenic imbalance may underlie differences in uteroplacental vascularization and fetoplacental development within the litter [35]. It would be interesting to identify whether there are alterations in angiogenic factor expression that explain the differences in placental morphology of the lightest versus heaviest fetuses of the two sexes in the litter.

### Placental mitochondrial function

Previous studies have shown that in human pregnancies associated with FGR, although mRNA expression of ETS complexes (II, III, and IV) is lower, there is higher mitochondrial DNA (mtDNA) content and higher oxygen consumption related to mitochondrial bioenergetics in the placenta [36]. Similarly, FGR induced by maternal caloric restriction in rats is associated with augmented mitochondrial biogenesis, as evidenced by the increased expression of PGC-1α, NRF1, and *Tfam*, as well as elevated complex I and IV dependent respiration in the placenta [21]. These data suggest that a common response of the placenta to try and meet the genetically determined demands of the fetus for growth during gestation involves the modulation of placental mitochondrial respiratory capacity. This study supports this notion and shows that the functional characteristics of placental mitochondria also adapt with natural variations of fetal growth in normal pregnancy. In females, expression of the biogenesis promoter gene *Nrf1*, fission regulator genes *Drp1* and *Fis1*, and mitochondrial complex I protein were lower, yet complex I LEAK and complex I + II OXPHOS rates were greater in the placenta supporting the lightest compared to the heaviest females. Whereas in males, biogenesis gene *Tfam* was greater, yet mitochondrial complexes III and V proteins were lower and LEAK and OXPHOS rates were not different in the placenta supporting the lightest compared to the heaviest males. Therefore, for both sexes, the placentas of the lightest fetuses appear to increase mitochondrial respiratory efficiency (as there were reduced mitochondrial complexes yet unaltered or increased respiration), although the underlying mechanisms and extent to which this may occur are different for females and males. Indeed, our results suggest that mitochondria in the placenta sustaining the lightest female fetuses in the litter are more responsive to adaptative mechanisms, as they exhibited increased mitochondrial respiration rates. This enhanced adaptive response may have been beneficial in providing the energy to sustain the expression of glucose transporters for the lightest fetuses. Previous work has demonstrated there is a reduced abundance of all mitochondrial complexes and lower OXPHOS respiration rates in placental trophoblast from obese women who deliver high birth weight babies [37]. Moreover, mitochondrial complex activity is also decreased in the placenta from women with pre-pregnancy obesity or pre-gestational diabetes who have LGA babies [38]. Thus, the natural variation in intra-litter placental mitochondrial function in the current study is likely the outcome of adaptive responses in operation for both the lightest and heaviest fetuses.

In the lightest female, but not lightest males, there was lower CLPP protein abundance when compared to the heaviest female fetuses of the litter. CLPP is also decreased along with mitochondrial complex abundance in the placenta of preeclamptic women delivering FGR babies [39]. The biological relevance of the difference in placental CLPP level between the lightest and heaviest fetuses is currently unknown. However, differences in placental CLPP protein may be particularly relevant for the outcome of female and male fetuses if the gestation is challenged, such as by a hypoxic or nutritional stimulus [24].

### Placental sex steroid handling

The placental expression of key steroid synthetic enzyme genes was differentially altered between the lightest and heaviest females only. The greater *Cyp11a1* while lower *Cyp17a1* gene expression in the lightest compared to the heaviest female fetus, would be expected to enhance the synthesis of the steroid hormone precursor pregnenolone, but also limit the synthesis of sex steroids. Indeed, other work on the placenta has shown that mRNA expression of steroidogenic enzymes is associated with the synthesis of steroid hormones [40]. CYP11A1 and CYP17A1 proteins are both cytochrome P450 monooxygenases located in the mitochondrial membrane that use oxygen for steroidogenesis, and changes in their expression may have relevance for understanding the greater rate of oxygen consumption in LEAK state for the placenta of the lightest versus heaviest females. Other work has shown that CYP11A1 protein is upregulated in the placenta of women with preeclampsia and overexpression of CYP11A1 protein in human trophoblast cells reduces proliferation and induces apoptosis [41, 42]. In addition, in vitro studies using cell lines have implicated an important role of CYP17A1 in placental estrogen production [43]. Thus, further studies would benefit from quantifying steroid hormone levels in the placenta. The expression of the androgen receptor gene was greater in the placenta of the lightest compared to the heaviest males; a difference not seen by doing the same comparison in females. These data suggest enhanced sensitivity of the lightest male placenta to androgens, namely testosterone, which can be produced by the fetal testes from approximately day 12–13 of mouse [44]. Interestingly, in rats, elevated testosterone levels disrupt the number and structure of mitochondria in the placenta and decrease fetal weight [45, 46]. Additionally, DHT (5α-reduced metabolite of testosterone) and insulin treatment of rats induces mitochondrial damage and an imbalance between oxidative and anti-oxidative stress responses in the placenta in association with FGR [47]. Thus, differences in steroid production and signaling are likely involved in the underlying alterations in placental morphology and mitochondrial functional capacity supporting the lightest fetus of each sex.

### Placental signaling pathways

The mechanisms underlying the differences in intra-litter placental mitochondrial function for each fetal sex are unknown. However, intra-litter differences in placental morphology and mitochondrial functional capacity likely stem from variations in the abundance of AMPK, AKT, and PPARγ proteins between the lightest and heaviest female and male fetuses [16]. Increased levels of AMPK protein in the placenta were seen for both the lightest females and lightest males compared to their heaviest counterparts. AMPK is activated by an increase in the AMP to ATP ratio and hence, is reflective of a decline in energy status. In turn, AMPK activates metabolic enzymes that allow cells to switch on catabolic pathways that generate ATP, including glycolysis and fatty acid β-oxidation [48]. We did not observe differences in placental FAO between the lightest and heaviest of each fetal sex within the litter. However, it would be beneficial to assess glycolysis and glycolytic enzyme expression in the placenta to assess whether there are intra-litter differences for females or males. In addition to its role in energy sensing, AMPK regulates placental trophoblast differentiation, proliferation, and nutrient transport [49]. Moreover, AMPK in the placenta has been linked to maternal vascular responses and changes in placental morphology and fetal growth in hypoxic pregnancy [8, 50]. Interestingly, compared to the heaviest fetuses, the magnitude of increase in placental AMPK protein was greatest for the lightest females (increased by 67% in the lightest females and increased by 41%, in the lightest males). Whether this may relate to the observation of altered placental morphology in only the lightest females requires further study.

Activation of AKT (phosphorylated AKT protein) was lower in the Lz of the lightest compared to heaviest male fetuses only. The AKT–mTOR signaling pathway plays a crucial role in the regulation of placental transport function and it was shown to be upregulated in pregnancies from obese women delivering LGA babies [51] and down-regulated in placentas from SGA/FGR babies [52]. Indeed, mTOR signaling is a positive regulator of genes encoding ETS proteins and mitochondria respiratory function [53]. Moreover, placental trophoblast-specific loss of phosphoinositol-3 kinase (PI3K) signaling, which is upstream of AKT, leads to FGR in mice [54]. In line with the reduced AKT activation, only the lightest males presented lower glucose transporter gene expression (*Slc2a1*, encodes *GLUT1*) in their placenta, when compared to the heaviest males. Insulin is a major fetal growth factor that signals via AKT to mediate its metabolic effects. Reduced activation of AKT in the placenta of the lightest males may therefore reflect reduced fetal insulin signaling to the placenta [55]. These data may also reinforce the idea that the placentas of females and males execute their molecular mechanisms to best support fetal growth and development. Genetic studies in mice have shown that mutations in genes encoding glucose transporter proteins result in FGR [56]. Moreover, in humans, placental GLUT1 protein is down-regulated in preeclampsia and this might play a role in the coincident development of FGR [57]. Conversely, women with insulin-dependent diabetes who have an increased incidence of LGA show increased GLUT1 protein expression and a higher mediated uptake of D-glucose by the placenta [58]. Since glucose is the most important energetic substrate for fetal growth, lower expression of the placental *Slc2a1* gene in the lightest relative to the heavier male fetus may explain their fetal growth discrepancy.

Differences in placental phenotype for each fetal sex may also relate to changes in PPARγ protein abundance; PPARγ was greater in the lightest females, but lower in the lightest males compared to their respective heaviest counterparts. In humans, PPARγ expression was found to be reduced in the placenta of SGA fetuses and to associate positively with fetal and placental weights within this subpopulation [59]. In addition, PPARγ modulates the expression of amino acid transporters LAT1 and LAT2 (encoded by *Slc7a5* and *Slc3a2* genes*,* respectively) and plays a key role in the control of fetal growth [60]. PPARγ is also important for the regulation of lipid uptake and metabolism and regulates mitochondrial function via multiple routes [61]. While no differences were detected in the placental expression of amino acid and fatty acid transporters, changes in PPARγ protein could in part mediate the differences in mitochondrial respiratory capacity and regulatory factor expression between lightest and heaviest fetuses in our study. PPARγ is a transcription factor that can be modulated by numerous signals, including hormonal/growth factor signaling pathways, inflammatory/stress signaling pathways, and cellular metabolite levels [62]. Hence, alterations in PPARγ abundance in the placenta of the lightest females and the lightest males likely reflect variations in the metabolic and hormonal environment of those fetuses relative to their heaviest counterparts within the litter.

Differences in placental phenotypes and signaling pathways, like PPARγ with fetal weight in the two sexes, may be related to imprinted genes. Other work which did not assess the fetal sexes separately and also instead assessed placental phenotype between the lightest and heaviest placentas within the litter found altered expression of imprinted genes, including *Igf2* [3]. Moreover, without considering intra-litter weight differences, the expression of several imprinted genes differs between the two sexes, such as *paternally expressed 3 (Peg3), zinc finger, imprinted 1 (Zim1), pleiomorphic adenoma gene-like 1 (Igf2), imprinted maternally expressed transcript (H19),* and *Zac1* [63]. Finally, this study has also demonstrated that disrupting the imprinting region ICR1 (which controls expression of the reciprocally expressed imprinted genes, *Igf2* and *H19*) affects placental endocrine capacity in females and males differently [64]. However, the expression of *Igf2P0* and *Dlk1*, imprinted genes reported to be linked with feto-placental growth and PPARγ, respectively [31, 65, 66] did not differ between the lightest and heaviest fetuses of either sex. The expression of *Slc38a4*, an imprinted gene that was measured to inform on alterations in placental amino acid transport capacity, also did not vary between the lightest and heaviest fetuses of each sex. Thus, further work is required to ascertain the specific contribution of imprinted genes to differences in placental phenotype between the lightest and heaviest fetuses for each sex in the litter.

### Placental phenotype and fetal sex comparisons

Previous work has shown there are ontogenic changes in placental Lz morphology, function, mitochondrial respiration, and mitochondrial-related regulators that support the growing demands of the fetus during normal late mouse pregnancy [24]. In this study, the retrospective assessment showed that the heaviest males and their placentas were heavier than the heaviest females within the litter, although no differences were found in placental morphology, mitochondrial respiratory capacity (respiration rate or mitochondrial-related gene expression), or transport/hormone genes between them. In contrast, the lightest males and their placentas did not differ in weight when compared to the lightest females, yet they varied in the placental expression of nutrient transporters, steroidogenesis genes, mitochondrial respiration (complex I OXPHOS rate), and mitochondrial-related gene expression. These data suggest that male and female fetuses may differentially execute a placental response depending on their ability to reach (lightest fetuses) or supersede (heaviest fetuses) their genetic growth potential. Assessing fetal hormone and nutrient/metabolite levels in the lightest and heaviest fetuses of both sexes in the litter may provide some insight into the mechanisms underlying the differences seen in the placenta. The possible involvement of fetal hormone and nutrient/metabolites in mediating adaptations in the placenta could be tested using fetal-specific manipulations [54], but identification of the precise underlying mechanisms may be highly challenging. Future work would also benefit from assessing the timing of changes occurring in the placenta relative to the pattern of fetal growth for the males and females within the litter. This will help to identify whether fetal weight discrepancies within the litter are the cause or consequence of placental adaptations that started during early mouse pregnancy.

### Study strengths and limitations

Our study has clear strengths. It provides a comprehensive analysis of the structural, functional, mitochondrial, and molecular differences in the isolated transport Lz for the lightest and heaviest fetuses of each sex in litters of normal, healthy pregnant mice. However, the Lz is composed of numerous cell types, and the contribution of each cell population to the specific placental alterations seen is unknown. We also do not know if there are alterations in the endocrine junctional zone, which is also important for the support of fetal growth [67]. Moreover, as we did not record the uterine position of the individual fetuses, whether placental changes are driving alterations in fetal growth based on maternal supply differences secondary to perfusion/implantation variations for each sex could not be ascertained. Indeed, differences in the placenta could also be influenced by the sex of adjacent fetuses and variations in litter size [35, 68, 69], which would need to be addressed using much larger sample sizes to ensure there is sufficient statistical power. We also employed gene expression analysis as a surrogate measurement of placental transport and endocrine capacity, and further work would benefit from undertaking *in vivo* transport assessments [70] or hormone secretion assays [71]. Finally, it is challenging to extrapolate our findings from a polytocous animal species to human pregnancy.

## Summary

In summary, our data show that the placental transport zone (Lz) adopts different strategies, at the level of morphology, nutrient transport, steroid handling, and mitochondrial function to support the growth of the lightest and the heaviest fetuses within the litter in normal physiological mouse pregnancy. These adaptations are likely mediated via metabolic (e.g. lipids, energy status) and endocrine cues (insulin, sex steroids) within the fetus that trigger signaling pathways (e.g. AMPK, PPARγ, AKT) in the placenta, initiating pleiotropic effects. Further work is required to test the mechanisms underlying phenotypic differences in the placenta and to ascertain the relevance of our findings for pregnancies with adverse conditions, such as maternal malnutrition, obesity, or reduced oxygen availability where the maternal ability to provide resources to the fetus for growth are constrained. From a clinical perspective, our data may be important for understanding the pathways leading to placental insufficiency and fetuses not reaching (FGR/SGA) or exceeding their genetically determined growth potential (LGA). They may also have significance in understanding the discordance in weight and perinatal outcomes between babies of multiple gestations in women. Moreover, since the spectrum of pregnancy outcomes and the factors causally involved are likely to be many, determining how placental phenotype interacts with the weight of female or male fetuses within normal mouse litter may be useful to the design of sex-specific therapeutic agents to improve pregnancy outcomes in humans. This is highly relevant given the profound impacts of fetal growth and pregnancy complications on the immediate and life-long health of the child.

## Authors’ contribution

ESP, JLT, and ANSP designed the study. ESP, JLT, and DPC performed the experiments and analyzed and graphed the data. ESP and ANSP wrote the paper. All authors contributed to data interpretation and performed final editing checks and approved the final manuscript.

## Data availability

All data are available upon reasonable request.

## Conflicts of interest

The authors have declare that no conflict of interest exists.

## Supplementary Material

Supplementary_Table_1_ioac056
