# Supplementary material for: Placental structure, function, and mitochondrial phenotype relate to fetal size in each fetal sex in mice
Source: Biol Reprod. 2022 Mar 16;106(6):1292–311. doi: 10.1093/biolre/ioac056 (PMC9327737; doi:10.1093/biolre/ioac056)
Supplement: Supplementary_Table_1_ioac056 [file supplementary_table_1_ioac056.docx]

**Supplementary Table 1**. Litter size and sex percentages for each pregnancy.

Data expressed as Mean ± SEM

| Litter ID | Litter size | % Females | % Males |
| --- | --- | --- | --- |
| 4119 | 9 | 33.3 | 66.7 |
| 4120 | 10 | 60.0 | 40.0 |
| 4121 | 9 | 66.7 | 33.3 |
| 4122 | 7 | 28.6 | 71.4 |
| 4123 | 9 | 66.7 | 33.3 |
| 4128 | 8 | 37.5 | 62.5 |
| 4129 | 8 | 50.0 | 50.0 |
| 4130 | 8 | 50.0 | 50.0 |
| 4309 | 9 | 44.4 | 55.6 |
| 4320 | 7 | 57.0 | 42.9 |
| 4321 | 6 | 33.3 | 66.7 |
| 4329 | 8 | 50.0 | 50.0 |
| 4340 | 6 | 50.0 | 50.0 |
| Average | 8.00 ± 0.34 | 48.3 ± 3.5 | 51.7 ± 3.5 |
